# Astragaloside positively regulated osteogenic differentiation of pre-osteoblast MC3T3-E1 through PI3K/Akt signaling pathway

**DOI:** 10.1186/s13018-021-02690-1

**Published:** 2021-10-07

**Authors:** Wei Bing Jing, Hongjuan Ji, Rui Jiang, Jinlong Wang

**Affiliations:** 1 Department of Orthopedics, The People’s Hospital of Danyang, Affiliated Danyang Hospital of Nantong University, Danyang, 212300 Jiangsu Province China; 2 Department of Orthopedics, Huai’an Second People’s Hospital, The Affiliated Huaian Hospital of Xuzhou Medical University, Huai’an, China; 3 Department of Orthopedics, Lianshui County People’s Hospital, 6 Hongri Road, Huai’an, 223400 Jiangsu P.R. China; 4 Department of Orthopedics, Hongze District People’s Hospital, 102 Dongfeng Road, Hongze District, Huai’an, 223100 Jiangsu Province China

**Keywords:** Astragaloside, PI3K/Akt pathway, MC3T3-E1 cells, Osteoporosis

## Abstract

**Background:**

Osteoporosis is a widespread chronic disease characterized by low bone density. There is currently no gold standard treatment for osteoporosis. The aim of this study was to explore the role and mechanism of Astragaloside on osteogenic differentiation of MC3T3-E1 cells.

**Methods:**

MC3T3-E1 cells were divided into control and different dose of Astragaloside (10, 20, 40, 50, and 60 μg/ml). Then, ALP and ARS staining were performed to identify the effects of Astragaloside for early and late osteogenic capacity of MC3T3-E1 cells, respectively. Real-time PCR and western blot were performed to assess the ALP, OCN, and OSX expression. PI3K/Akt signaling pathway molecules were then assessed by Western blot. Finally, PI3K inhibitor, LY294002, was implemented to assess the mechanism of Astragaloside in promoting osteogenic differentiation of MC3T3-E1 cells.

**Results:**

Astragaloside significantly increased the cell viability than the control group. Moreover, Astragaloside enhanced the ALP activity and calcium deposition than the control groups. Compared with the control group, Astragaloside increased the ALP, OCN, and OSX expression in a dose-response manner. Western blot assay further confirmed the real-time PCR results. Astragaloside could significantly increase the p-PI3K and p-Akt expression than the control group. LY294002 partially reversed the promotion effects of Astragaloside on osteogenic differentiation of MC3T3-E1 cells. LY294002 partially reversed the promotion effects of Astragaloside on ALP, OCN, and OSX of MC3T3-E1 cells.

**Conclusion:**

The present study suggested that Astragaloside promoted osteogenic differentiation of MC3T3-E1 cells through regulating PI3K/Akt signaling pathway.

## Background

Osteoporosis is a common systemic bone metabolism disease [1, 2]. It is characterized by reduced bone mass, bone strength, and damage to the bone microstructure, which directly leads to an increase in bone fragility, so the incidence of fractures in patients with osteoporosis increases [3]. As a global health problem, the incidence of osteoporosis has been high [4]. In addition, women face a sharp decline in sex hormones after aging, and the incidence of spondylosis is much higher than that of men [5]. According to statistics, there are 40% of white postmenopausal women suffering from the adverse effects of osteoporosis, and with the development trend of the global population aging, this proportion will keep rising [5]. Among the adverse clinical consequences of osteoporosis, hip fractures and vertebral body fractures are the most serious, and the mortality rate can be as high as 20% after the onset of the disease [6].

At present, the most common clinical treatment plan for osteoporosis is combination medication, which is mainly concentrated and limited to anti-bone resorption and/or promote bone formation [7]. However, most drugs have low sensitivity and many side effects in the population and can even cause complications such as osteonecrosis of the jaw, esophageal irritation, or hypocalcemia [8]. Therefore, looking for more suitable treatment targets, more effective, efficient, safe, and more compliant treatment options are currently urgent problems to be solved.

The pathogenesis of osteoporosis is mainly the disorder of bone homeostasis, that is, the imbalance between osteogenesis and osteoclastogenesis [9]. DangguiBuxue Tang (DBT) is an ancient Chinese herbal decoction that traditionally used to treat menstrual anemia [10]. DBT is used to tonify the liver and kidney, promoting blood flow and removing blood stasis, and strengthen tendons and bones [11].

Astragalus is a popular traditional Chinese medicine commonly used as a constituent in tonic herbal preparations [12]. Astragaloside is the dominant active component of Astragalus. Previous study found that Astragaloside could delay cartilage degeneration [13]. However, whether Astragaloside has a beneficial role in promoting osteogenesis was unknown.

PI3K/Akt signaling pathway is crucial in cell proliferation, differentiation, and adaptation [14]. Previous study found that PI3K/Akt signaling pathway is crucial for osteoblast differentiation in multiple stem cells [15–17]. Thus, we hypothesized that Astragaloside stimulated osteogenic differentiation of MC3T3-E1 cells through PI3K/Akt signaling pathway.

This study aimed to explore whether Astragaloside promoted osteogenic differentiation of pre-osteoblast MC3T3-E1 cells through PI3K/Akt signaling pathway.

## Material and methods

### Cell source and osteogenic induction

MC3T3-E1 cells were obtained from the cell library of Shanghai Chinese Academy of Sciences (Shanghai, China). Astragaloside was purchased from Xi’an Sobeo Pharmaceutical Technology Company, Limited (purity above 98%, Xi’an, China). Astragaloside was dissolved into DMSO, and the final DMSO concentration did not exceed 0.1% (v/v). MC3T3-E1 was cultured into DMEM/F12 medium supplemented with 10% FBS and 1% penicillin-streptomycin at 37 °C and 5% CO_2_. The osteogenic differentiation media comprised of dexamethasone (100 nM), β-glycerophosphate (10 mM), and l-ascorbate (0.2 mM). MC3T3-E1 were seeded at a density of 5 × 10^5^ cells/well in six-well plates. When cells reached approximately 80% confluence, cells were changed to osteogenic differentiation media. Medium was replaced with fresh one every second day.

### Bioinformatic analysis of Astragaloside

STITCH database (http://stitch.embl.de/) was searched for the potential target genes of Astragaloside. The minimum required interaction score was set as 0.900 and hide the disconnected nodes. Then, these target genes were enriched for gene ontology and KEGG pathway analyses. Gene Ontology (GO) and Kyoto Encyclopedia of Genes and Genomes (KEGG) pathway analysis and visualization were performed using R (Version 3.5.0).

### CCK-8 assay

MC3T3-E1 were incubated at 96 wells at a density of 1×10^4^/ml. Then, MC3T3-E1 cells were incubated with different concentration of Astragaloside. After 3 days of culture, the medium was discharged and 10 μl CCK8 reagents was added. The plates were then incubated for another 2 h. Absorbance at 450 nm was measured using a microplate reader (BioTek microplate reader). The cell viability (%) normalized to the control group was calculated.

### Alp

MC3T3-E1 cells were incubated in six-well plates. Then, osteogenic induction medium or osteogenic induction medium containing Astragaloside were added in the plates. ALP staining was performed after 7 days in culture. In brief, medium was removed and washed with PBS for three times. Then, the PBS was discharged and fixed with 4% paraformaldehyde for 15 min. Staining was performed using NBT/BCIP solution until satisfied. Reactions were terminated by the addition of distilled water.

### ARS

Medium was removed and washed with PBS for three times. Then, 4% of paraformaldehyde was added for 15 min to fix the MC3T3-E1 cells. Appropriate amount of Alizarin Red dye was added and incubate at room temperature for 30 min. Alizarin Red dye was then discarded and washed with PBS for three times. A light microscope (Olympus) was employed to take pictures. The stained cells were then eluted with 10% cetylpyridinium chloride. After full dissolution, solution absorbance was measured with a microplate reader (BioTek microplate reader) at 405 nm.

### Real-time PCR

MC3T3-E1 cells were washed with PBS for three times, and Trizol (Invitrogen, Carlsbad, CA, USA) was added. Chloroform was added to the suspension to give a ratio of 5∶1 of Trizol to chloroform (v/v). After centrifugation, the RNA-containing aqueous phase was aspirated, and the A260/A280 ratio of all RNA samples was >2.0 as measured by Nanodrop. cDNA was synthesized using the PrimeScript RT reagent kit (Perfect Real Time) (TaKaRa, #RR037A). The qRT-PCR was performed using TB Green® Premix Ex Taq™ (Takara, China). The housekeeping gene GAPDH was used as an internal reference. Primers were as follows: ALP: 5′-AACAGACAAGCAACCCAAAC-3′; 5′-TAACCCAACGGGCAGAAA-3′;

OSX: 5′-CAAATACCC AGATGCTGGGC-3′; 5′-TCCTGGCTGTCCACATGGAC-3′;

OCN: 5′-CAGACCTAGCAGACACCATGAG-3′; 5′-CGTCCATACTTTCGAGGCAG-3′;

GAPDH: 5′-CCCCGCTACTCCTCCTCCTAAG-3′; 5′-TCCACGACCAGTTGTCCATTCC-3′;

### Western blot

The plates were washed with PBS three times. Total protein was extracted using a lysis buffer containing PMSF and RIPA (PMSF: RIPA=1: 99). Protein concentration was measured by BCA assay. Extracted proteins were separated by SDS-PAGE (12% acrylamide) and blotted onto PVDF membranes. Blots were blocked with 5% nonfat dry milk for 2 h. PVDF membranes were then incubated for primary antibodies overnight at 4°C. The next day, PVDF membranes were incubated with a second antibody labeled by HRP at room temperature for 1 h. Protein bands were detected using chemiluminescence, and ImageJ was used to quantify Western blot band densities.

### Statistical analysis

Results were presented as mean ± standard deviation (SD). All experimental data were analyzed using one-way analyses of variance (ANOVA) with Bonferroni’s post hoc test using SPSS 21.0 (IBM Corp., Armonk, NY, USA). *P* value less than 0.05 was identified as statistically significant. All experiments were performed more than three times.

## Results

### Astragaloside increased cell viability of MC3T3-E1 cells

**Fig. 1:**
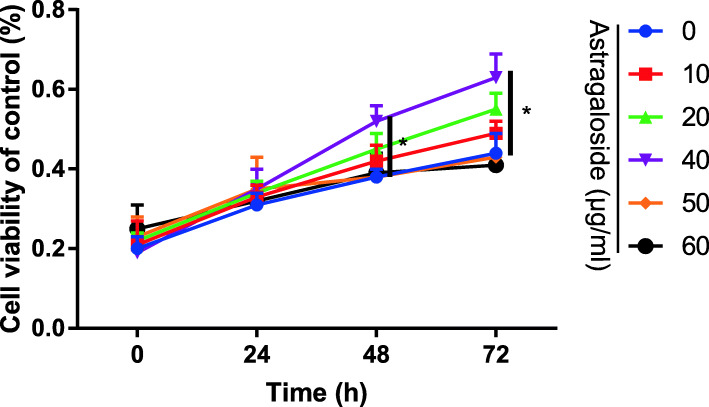
CCK-8 assay to assess the Astragaloside on cell viability at 24, 48, and 72 h

CCK-8 assay was performed to evaluate cell viability. Astragaloside increased cell viability in a dose-dependent manner in cultured MC3T3-E1 cells compared to the control group. The most effective was the concentration of Astragaloside at 40 μg/Ml (Fig. 1).

### Astragaloside increased osteogenic differentiation of MC3T3-E1 cells

**Fig. 2:**
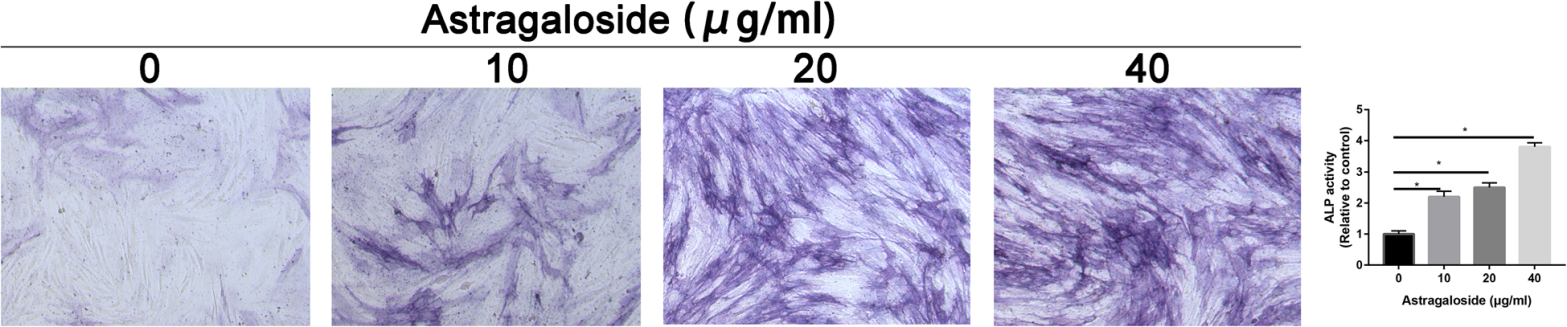
ALP staining in control and Astragaloside-treated MC3T3-E1 cells

**Fig. 3:**
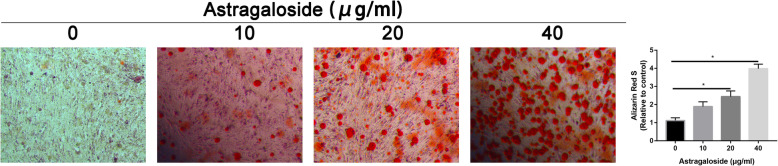
ARS staining in control and Astragaloside-treated MC3T3-E1 cells

To further validate whether Astragaloside could govern the osteogenic differentiation of MC3T3-E1 cells, ALP (Fig. 2) and ARS (Fig. 3) stainings were performed. Results found that Astragaloside increased ALP activity and calcium deposition of MC3T3-E1 cells in a dose-response manner. When the Astragaloside concentration was 40 μg/mL, the ALP activity and calcium deposition was significantly higher than that of other concentration group.

### Astragaloside significantly increased osteogenic differentiation markers expression

In order to identify Astragaloside has a positive role in promoting osteogenic differentiation of MC3T3-E1 cells, real-time PCR was performed to real the explore the osteoblast markers expression.

**Fig. 4:**
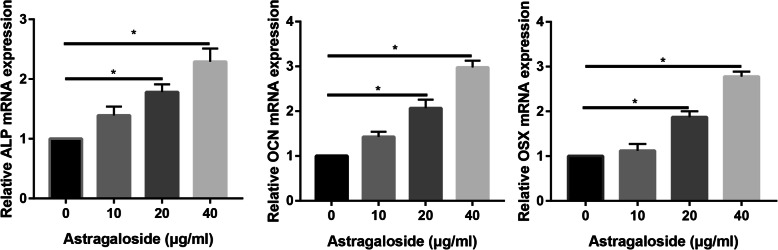
Real-time PCR assay to assess the osteogenic markers in control and Astragaloside-treated MC3T3-E1 cells

As illustrated in Fig. 4, the ALP, OCN, and OSX mRNA increased 3.52-fold after treatment with Astragaloside compared to the control group (*P* < 0.05). In addition, 50 μg/mL Astragaloside could even more strongly increase ALP, OCN, and OSX expression, which increased 5.67-fold compared to that of the control group, with statistically significant differences (*P* < 0.05).

**Fig. 5:**
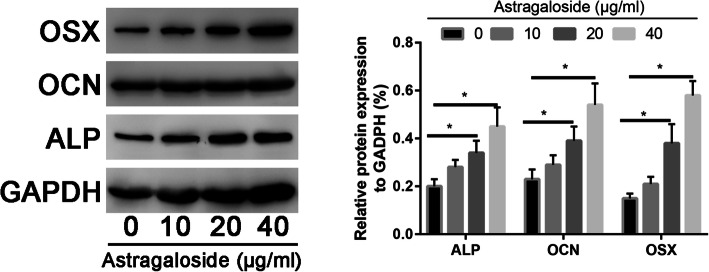
Western blot assay to assess the osteogenic markers in control and Astragaloside-treated MC3T3-E1 cells

Moreover, Western Blot results indicated that ALP, OCN, and OSX had the same expression pattern with the real-time PCR assays (Fig. 5).

### Bioinformatic analysis of Astragaloside

**Fig. 6:**
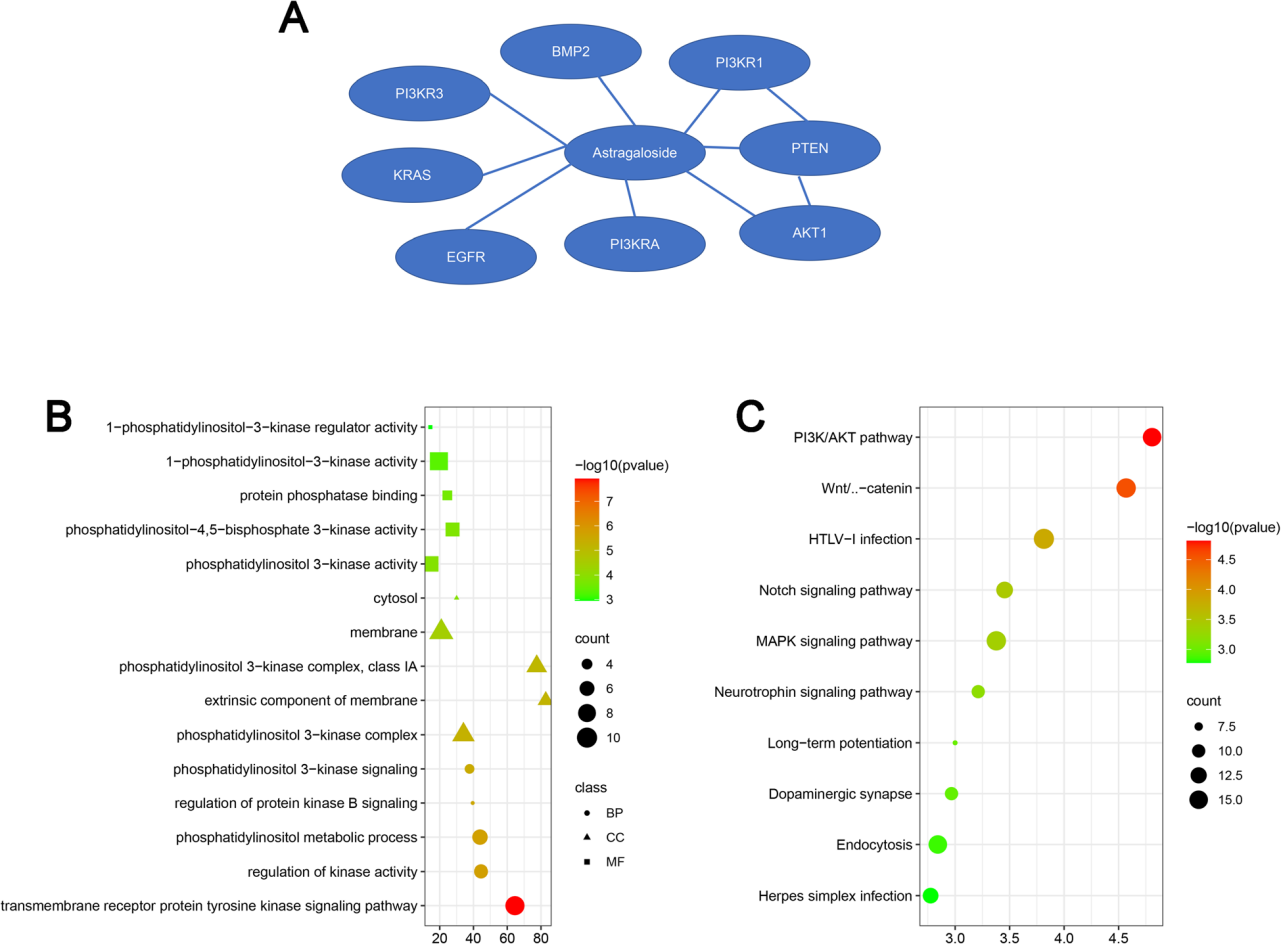
Bioinformatic analysis of the Astragaloside target genes. **a** Target genes of Astragaloside through search of the STITCH database; **b** Gene ontology of the target genes of Astragaloside; **c** KEGG pathway analyses of the target genes of Astragaloside

Through STITICH database, Astragaloside directly target with BMP2, PI3KR1, AKT1, PI3KRA, EGFR, KRAS, and PI3KR3 (Fig. 6 A). Target genes are mainly enriched in transmembrane receptor protein tyrosine kinase signaling pathway, regulation of kinase activity, phosphatidylinositol metabolic process, regulation of protein kinase B signaling, phosphatidylinositol 3-kinase signaling, phosphatidylinositol 3-kinase complex, extrinsic component of membrane, phosphatidylinositol 3-kinase complex, class IA, membrane, cytosol, phosphatidylinositol 3-kinase activity, phosphatidylinositol-4,5-bisphosphate 3-kinase activity, protein phosphatase binding, 1-phosphatidylinositol-3-kinase activity, and 1-phosphatidylinositol-3-kinase regulator activity (Fig. 6 B).

KEGG pathway were as follows: PI3K/AKT signaling pathway, Wnt/β-catenin signaling pathway, HTLV-I infection, Notch signaling pathway, MAPK signaling pathway, Neurotrophin signaling pathway, Long-term potentiation, Dopaminergic synapse, Endocytosis, and Herpes simplex infection (Fig. 6 C).

### Astragaloside increased osteogenic differentiation of MC3T3-E1 cells through activating PI3K/Akt signaling pathway

**Fig. 7:**
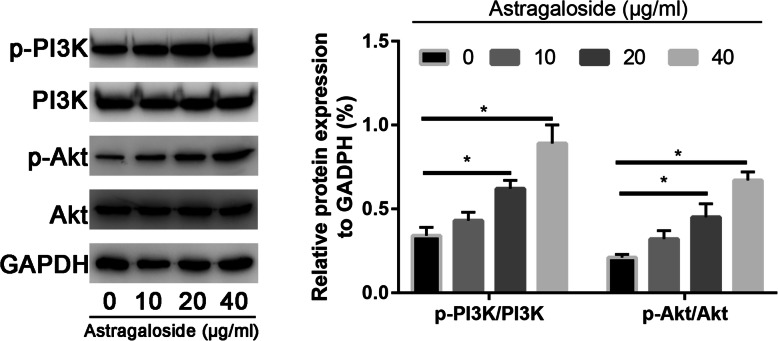
Western blot assay to assess the PI3K, p-PI3K, Akt, and p-Akt expression in control and Astragaloside-treated MC3T3-E1 cells

We further analyzed the expression of PI3K, p-PI3K, Akt and p-Akt expressions. It was found that Astragaloside increased the phosphorylation levels of PI3K (p-PI3K) and AKT (p-AKT) expression, without change of total PI3K and AKT expression. In addition, 40 μg/mL Astragaloside could even more strongly increase phosphorylation levels of PI3K (p-PI3K) and AKT (p-AKT) expression than other dose of Astragaloside (Fig. 7).

### LY294002 partially reversed the promotion effects of Astragaloside on osteogenic differentiation of MC3T3-E1 cells

**Fig. 8:**
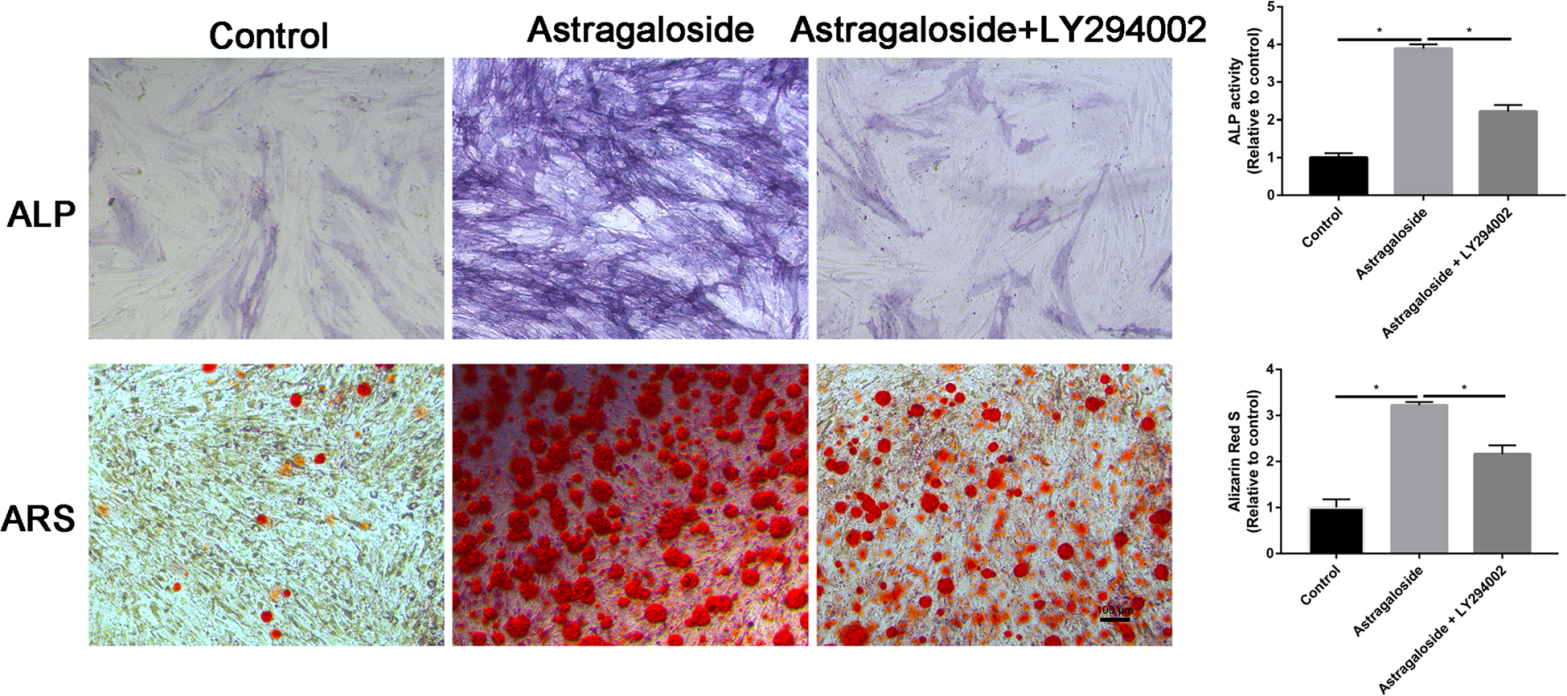
ALP and ARS staining of the MC3T3-E1 cells in control, Astragaloside, and Astragaloside + LY294002 groups

Astragaloside significantly enhanced the osteogenic differentiation of MC3T3-E1 cells. Pretreatment of LY294002, PI3K inhibitor, partially blocked the Astragaloside-induced osteoblastic activity of MC3T3-E1 cells (Fig. 8).

**Fig. 9:**
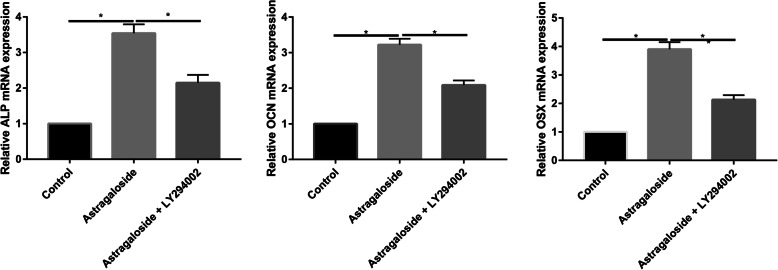
PCR assay to assess the osteogenic markers in control, Astragaloside, and Astragaloside + LY294002 groups

**Fig. 10:**
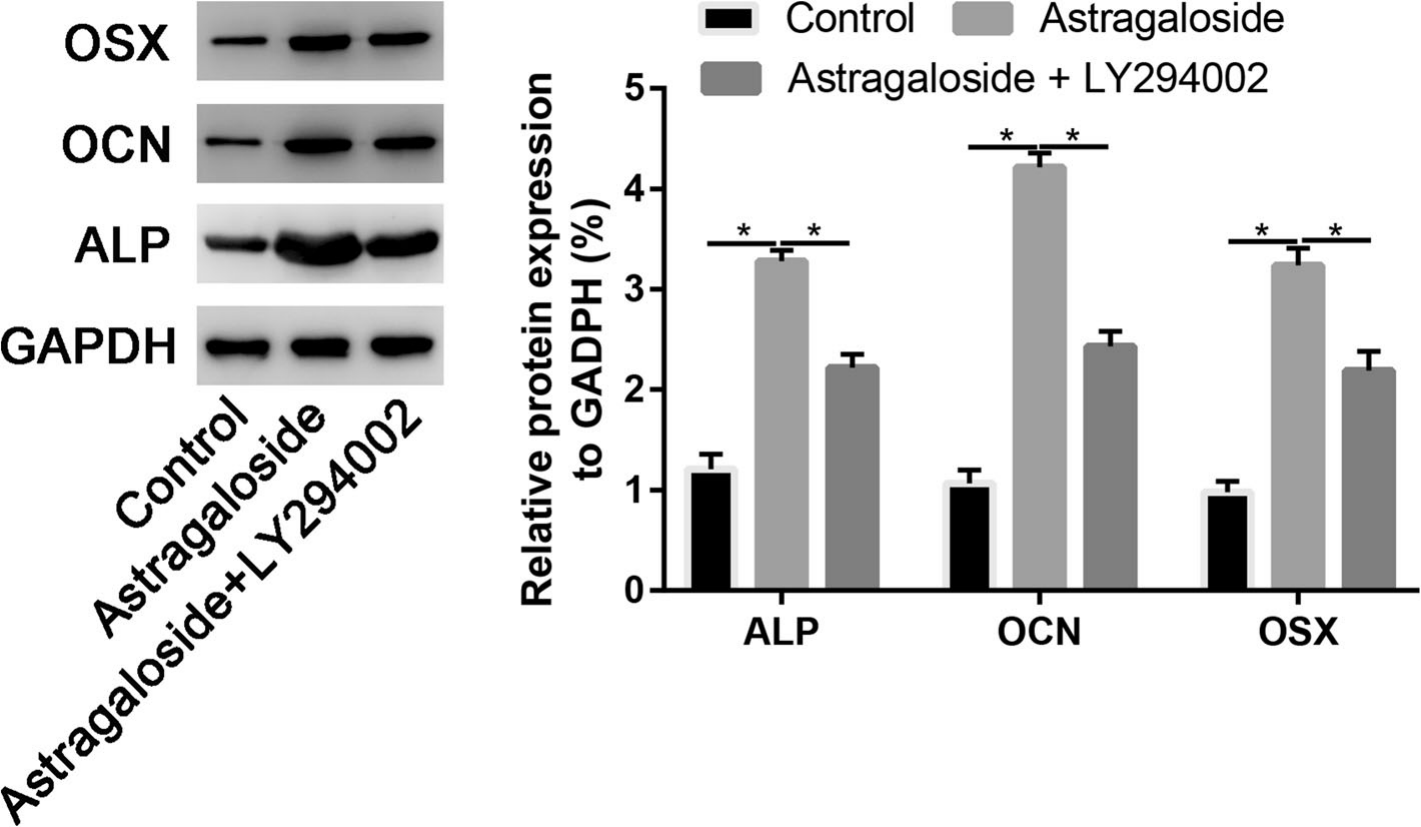
Western blot assay to assess the osteogenic markers in control, Astragaloside, and Astragaloside + LY294002 groups

We showed that inhibition of PI3K by LY294002 reduced ALP, Runx2, and OCN mRNA expression in osteoblasts (Fig. 9). Western blot analysis was in agreement with the quantitative real-time PCR (qRT-PCR) results, showing that the protein expression of ALP, Runx2, and OCN was downregulated in the LY294002 group (Fig. 10).

**Fig. 11:**
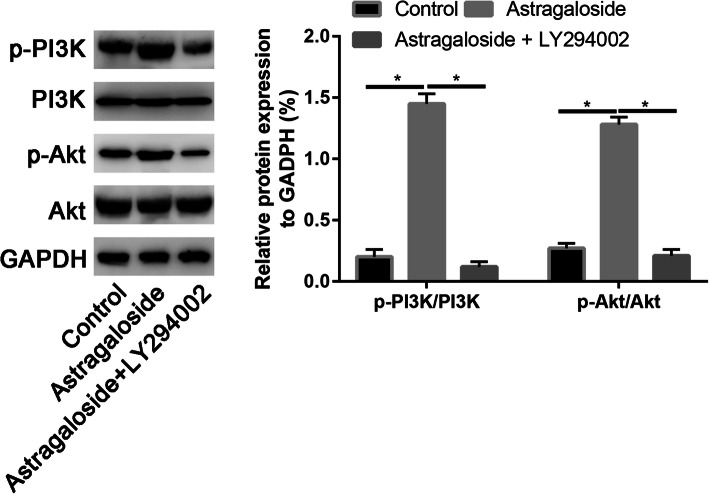
Western blot assay to assess the PI3K, p-PI3K, Akt, and p-Akt expression in control, Astragaloside, and Astragaloside + LY294002 groups

Western blot results found that LY294002 significantly decreased the p-PI3K and p-Akt expressions (Fig. 11).

## Discussion

In the present study, we further validated the protective effects of Astragaloside on bone formation. Our results, performed in vitro model and cultured MC3T3-E1 cells, provided assured evidence of the important role of Astragaloside in promoting osteogenic differentiation of MC3T3-E1 cells. The results that Astragaloside activated osteogenic process via promotion of PI3K/Akt pathway and indicated that Astragaloside could be served as a potential therapeutic strategy for bone-related disease.

Osteoporosis is a systemic disease that bone resorption and bone formation typically in a state of negative balance [18]. Deposition of calcium salts is a key step in the fracture healing [19]. In this study, we found that Astragaloside promoted osteogenic differentiation of pre-osteoblast MC3T3-E1 cells in a dose-response manner. The strength of new bone tissue is closely related to the degree of matrix calcification [20]. Alkaline phosphatase is a specific marker of early osteogenic differentiation of osteoblasts [21]. Astragaloside significantly increased the ALP activity than control group, which suggested that Astragaloside has a promotion role of bone repair. Astragaloside was extracted from traditional Chinese medicinal plant astragalus membranaceus [22]. Astragaloside possess antioxidant, anti-inflammatory, anti-diabetes, anti-hypertensive, anti-asthma, and anti-fibrotic properties [23–25].

The PI3K/Akt pathway is closely related to bone metabolism [26]. Many signaling molecules selectively activate osteoblasts P13K/Akt pathway and finally exert their specific effects [27]. Activating the PI3K/Akt pathway can promote the proliferation and differentiation of osteoblasts [28]. RNA sequencing analysis demonstrated that the P13K/Akt pathway was involved in bone regeneration. Thus, we selected P13K/Akt for further study. RUNX2 is the earliest and most specific marker gene in the process of bone formation. RUNX2 also serves as a marker for osteoblasts differentiation [29]. The RUNX2 homozygous knockout mice correlated with rib aplasia [30, 31]. These finding remind us that RUNX2 is critical for bone formation. In this study, Astragaloside significantly increased the RUNX2 expression and finally promoted osteogenic differentiation of MC3T3-E1 cells.

However, the limitations of this study should not be ignored. Whether Astragaloside regulates other signaling pathways is worth exploring further. The present study is based on in vitro experiments, and the results require to be further verified by in vivo experiments. More studies are needed to further clarify the pharmacokinetic variation and drug metabolism.

## Conclusion

In conclusion, Astragaloside promoted osteogenic differentiation of MC3T3-E1 cells through regulating PI3K/Akt signaling pathway. This proposes Astragaloside as a key potential target for primary osteoporosis in the perspective of molecular medicine. Therefore, in future, detailed clinical or in vivo experiments should be performed to support the arguments presented in this study.

## Data Availability

All of the materials and data in our paper can be availably obtained by reasonable request for corresponding author.

## Abbreviations

DBT: DangguiBuxue Tang
GO: Gene ontology
KEGG: Kyoto Encyclopedia of Genes and Genomes
SD: Standard deviation
ANOVA: One-way analyses of variance
